# Equal Inheritance: Genome Management for Proliferating Parasites

**DOI:** 10.1371/journal.pbio.1001445

**Published:** 2012-12-11

**Authors:** Stephanie Huang

**Affiliations:** Freelance Science Writer, San Francisco, California, United States of America

**Figure:**
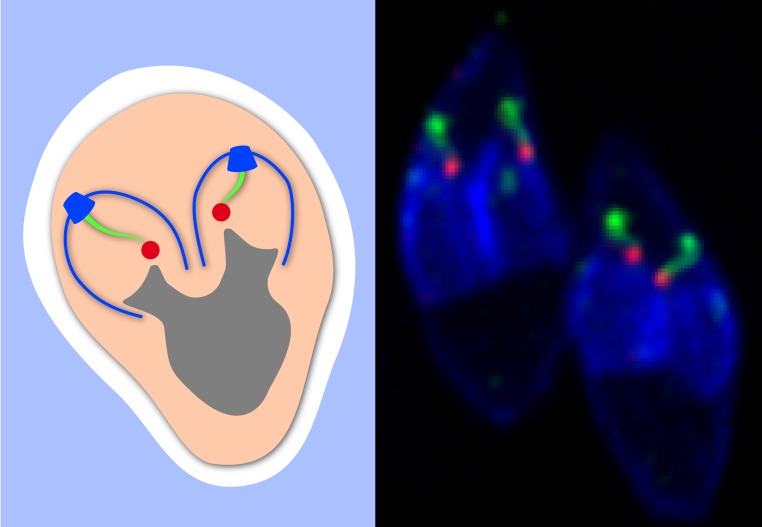
Parasite cell division depends on a fiber that once anchored the basal body of the flagellum in the algal ancestor. Here, you see the fiber (green), centrosomes (red), parasite daughter cells (blue), and nucleus (grey). The micrograph on the right depicts two *Toxoplasma gondii* parasites in division.

Of the 1,400 or so known human pathogen species, those belonging to the Apicomplexa phylum represent a special challenge to researchers. These single-celled protists cause some of the world's most prevalent parasitic diseases, including malaria (*Plasmodium falciparum*) and toxoplasmosis (*Toxoplasma gondii*). They are challenging to treat for two reasons. First, the parasites are eukaryotic and thus more similar to human cells than bacterial pathogens, making it difficult to find treatments that kill the parasite without harming human host cells. Second, the parasite cells reside within human host cells for much of their life cycle, evading detection by the host's immune system.

Apicomplexan parasites are named for a unique structure, the apical complex, which is present on the apical end of the parasite cell and enables the parasite to penetrate a host cell within seconds of contact. Once within a host cell, the parasite replicates and divides. *T. gondii* exhibit the simplest pattern of cell division, generally assembling two daughter cells within a mother cell, which then splits to produce the two daughter cells. Other species, like *P. falciparum*, may undergo multiple consecutive rounds of nuclear division, resulting in many copies of the parasite's DNA within one engorged cell, before splintering into as many as tens or thousands of daughter cells. The number of daughter cells can depend on the stage of the parasite and on which type of cell it has invaded. A *P. falciparum* parasite produces 10 to 20 daughter cells within a red blood cell and thousands of daughter cells within a liver cell.

This peculiar form of replication leads to interesting questions. With potentially tens or thousands of nuclei waiting in the replicating cell, how does the parasite know how many daughter cells it will need to accommodate the nuclei? And how does it distribute the nuclei to the daughter cells? In this issue of *PLOS Biology*, Maria Francia, Boris Striepen, and colleagues describe a fiber-like structure in *T. gondii* cells that appears to match each newly formed nucleus to a daughter cell, ensuring that each new parasite receives exactly one nucleus.

The team found that two proteins called TgSFA2 and TgSFA3 together formed two short fibers in the dividing parasite cell. Further microscopic work suggested these fibers may be important for cell division, because the SFA fibers appeared to form near the centrosome. In animal cells, centrosomes serve as organizing centers for the spindle, a set of microtubule protein fibers that reach into the mass of replicated chromosomes and pull the chromosomes apart into two equal sets during cell division. In fact, the team observed that the SFA fibers formed only after the centrosome split in two, a key event occurring at the start of cell division. The fibers eventually grew longer, extending away from the centrosomes and reaching into the developing daughter cells, ultimately terminating on their apical end.

Further experiments showed that the SFA fiber did not merely link the centrosome and developing daughter cells, but was required for the proper formation of the daughter cells. The researchers constructed mutant parasites that would stop producing either the TgSFA2 or TgSFA3 protein when treated with a drug. Without one of the SFA proteins, the mutant parasites swelled in size and accumulated multiple nuclei but were unable to divide successfully into daughter cells. Detailed microscopic imaging revealed that the daughter cells' pellicle—a structure that helps maintain the shape of the parasite cell and is the first structure to take shape in new cells—was poorly formed or failed to form at all. The team found that without the TgSFA2 or TgSFA3 protein, the developing daughter cells lacked key components of the pellicle, including microtubule fibers.

Taken together, these results show that SFA fibers may extend from centrosomes and help seed the nearby formation of a new daughter cell. During cell division, chromosomes are ultimately pulled towards one of the two centrosomes, thus rendering each centrosome a signpost for one complete set of genetic information. The SFA fibers, in other words, match each nucleus to a daughter cell by ensuring that a daughter cell forms only near each set of genetic information. Although Francia and her colleagues worked with *T. gondii* cells for their study, SFA proteins exist in other apicomplexan parasites and may work similarly in them as well. If so, the SFA fibers would provide an elegant explanation for how parasites such as *P. falciparum* can successfully allocate thousands of nuclei to its daughter cells.

Apicomplexan parasites evolved from algae, but, unlike their algal ancestors, most life cycle forms of the parasite do not carry flagella. The TgSFA2 and TgSFA3 proteins appear to have evolved from leftover flagellar components that anchored the flagella in the cell. The researchers propose that the parasites co-opted the ancient flagellar machinery to organize the microtubules and possibly also the apical complexes in the developing daughter cells. Looking into other ancient flagellar components may provide a starting point for further research on the SFA fibers and on the organization of the unique invasive structures of these parasites. A better understanding of the structures that enable the parasites to enter their host cells and divide within them may suggest ways to target these structures without causing collateral damage to human cells, safely breaking the parasitic cycle of development.

**Francia ME, Jordan CN, Patel JD, Sheiner L, Demerly JL, et al. (2012) Cell Division in Apicomplexan Parasites Is Organized by a Homolog of the Striated Rootlet Fiber of Algal Flagella. doi:10.1371/journal.pbio.1001444**

